# First Principles of Medicine

**Published:** 1844-05

**Authors:** 


Art. VI.—*First Principles of Medicine.* By Archibald Billing, M.D., A.M., Member of the Senate of the University of London, Fellow of the Royal College of Physicians, &c., &c., &c., &c. *First American from the Fourth London Edition.* Philadelphia: Lea & Blanchard. 1 vol. 8vo. pp. 304. 1842.

The age of industry, toil, and bustle, in which we live, seems to have communicated a spirit of enterprise, or it may be more proper to say a spirit of *ultraism*, to all the occupations of civilized life. The age of iron has passed, and has been succeeded by what may be emphatically called the age of steam. From him who rules the destinies of the mighty house of Hapsburg, through all the grades of political rulers, down to the most humble—all have an eye upon the operations of steam. The magnificent conceptions of the statesman are carried out by its power; the manufacturer, the agriculturist, and even the *printer*, have availed themselves of its advantages; and to judge of its mighty effects as a book-making power, it is hard to resist the idea that *pens* do not also go by steam.

The vast amount of books, both cheap and dear, good and entirely worthless, which are thrown off from the press and distributed upon this continent, is truly wonderful, and must form a strong characteristic feature of the age. It would be unreasonable to suppose, that, amid the excitement of this mania, the members of the medical profession should remain "lookers on," without contributing their mite. We accordingly find that our brethren, not to be behind the age, have industriously set themselves to work in manufacturing *new books* out of old ones, by re-moulding ideas that have been common to the profession from the days of Hippocrates to the present time; the consequence is that many of them are never read at all, or read to be consigned to that oblivion which they so justly merit.

Fortunately for the profession, however, there are many men of the present age, who have the talent and energy necessary to examine facts and form conclusions for themselves, and whose works are as refreshing to the student and seeker after truth, as the oasis to the wayworn traveller in the desert.

Such is the character of the work now before us, which deserves, for the many important truths it contains, and the manner in which they are told, a much more favorable reception than it has met with at the hands of the profession. It is the result of long, deep, and accurate thought, and has the merit of briefly stating *many* medical truths which are of the utmost importance to the practitioner.

For the purpose of showing the cast of mind and the determined spirit of inquiry of Dr. Billing, we will here introduce a short extract from the advertisement to the second edition of his book.

"I was originally led to publish this treatise by a recollection of the difficulties I had met with in the study of my profession, and by the hope that I might aid in removing them from the path of others.

"Upon commencing the study of medicine and surgery, after having become acquainted with the more minute physical sciences in the University, I was appalled to find it a complete chaos. Our family physician, really a man of great talent, and one of our professors, disheartened me by his answers. I inquired, 'What is fever?' Answer, of course, Cullen's definition. 'But what produces it?' 'Sometimes one thing, sometimes another: excessive cold or heat, or the effluvia from a person who has fever.' 'But what is the cause of the phenomena in the body?' 'Spasmodic contraction of the extreme vessels.' I could understand that cold might contract the extreme vessels, but I had been taught by the professor of Chemistry that caloric expanded everything. And, again, I did not see how effluvia produced spasm, nor how the spasm, even if it were produced, could make the skin extremely hot as well as cold. I was advised to read Cullen, and did so; but without finding the information I sought. Again I asked, 'When you give a dose of rhubarb or castor oil to stop a diarrhœa of many days' standing, how does it effect this object?' 'By clearing away the peccant matter.' 'But would not the diarrhœa scour away the peccant matter itself?' 'Not so well.' This did not satisfy me.

Neither could the surgeon clear up these points: 'for his part he did not pretend to understand physic.' I 'walked' the hospital at his elbow as dresser, and inquired, 'Why do you apply cold lotions to that inflammation?' 'To moderate the action of the vessels.' 'Inflammation, then, is over action of the vessels?' 'Yes.' 'Why do you apply that *astringent* (goulard or nitrate of silver) to that other inflammation?' 'To diminish the action of the vessels.' Now the *action* of the vessels being *contraction*, my logic did not enable me to comprehend him; so, after asking why he put a cold lotion or poultice on one inflamed part, and a warm poultice or fomentation on another, and being told I should find out by experience, I resolved on attempting to find out myself."

In giving a general view of the apparatus which supports the life of man, the author contends that the contraction of the heart only is muscular, and that of the arteries elastic and that the latter keep up a *constant* contractile force upon their contents, whether the amount of blood which they contain be great or small, keeping them constantly full, and at the same time exerting a force which is sufficient, during the temporary contractions of the heart, to cause the equability of the stream in the veins.

"It has been supposed," he adds, "that the circular fibres of the ARTERIES were muscular; that they contracted and relaxed at each pulse; and that the throb felt was caused by a dilatation of the artery. Those fibres are NOT MUSCULAR, but more approaching to a ligamentous tissue, firm, and, though elastic, not yielding to the force of the heart at each beat, but, on the contrary, preserving the CALIBRE of the artery UNIFORM, as may be seen by laying bare an artery in a living animal, or when the artery is exposed in an operation: it is LONGITUDINALLY that the arteries are STRETCHED at each injection from the heart, by which their capacity is increased; the consequence of which, from their being bound down in various places, is, that there is a SERPENTINE motion in the artery where it is at all loose. The fibres of the middle coat of the artery, being arranged circularly, allow of the separation laterally, and thus *accommodate* themselves to the *elongation* of the tube, whilst they *resist* its *dilatation*." (pp. 33, 34.)

This statement, we are told, has been confirmed by microscopical observations made by Schwann and Ehrenberg, in 1836; whereby this long-continued controversy has been set at rest, as regards the muscularity of the arteries.

It is also contended, and we think with much show of reason, that the heart exerts really a very feeble power in forcing the blood through the arteries and veins; for, upon the common hydrostatic principle of fluids finding their own level by the mere force of gravitation, we may account for the *greater* part of the force which is requisite to keep in motion so small an amount of fluid; the greater amount of blood, in all natural positions of the body, being below the level of the heart.

As we have promised ourselves to give nothing more than a passing notice of some of the general features of the book, we are compelled to omit many matters of interest; among others the action of the nerves upon the muscles and capillary arteries—giving barely this opinion of the Doctor's, that "all organic action is contraction, produced by nervous influence."

Upon the subject of animal heat, we have the following rather curious observations. These are the more remarkable, as they are almost identical with those since put forth by Liebig. Dr. Billing therefore seems to have anticipated the German professor:

"The ANIMAL HEAT has been accounted for in different ways by several ingenious physiologists: from the aggregate of their opinions and experiments I deduce, that *heat is extricated all over the frame;* in the *capillaries*, by the *action* of the *nerves* during the *change* of the *blood* from *scarlet* arterial to *purple* venous; and *also* whilst it is changing *in the lungs* from *purple* to *scarlet.*"

"There is a perpetual *deposition*, by the capillary system, of *new matter*, and decomposition of the *old*, all over the frame, influenced by the nerves: in other words, the galvanoid or electroid influence of the nerves, which occasions these depositions and decompositions, keeps up a slow combustion. In this decomposition there is a continual disengagement of carbon, which mixes with the blood returning to the heart at the time it changes from scarlet to purple; this *decomposition* being effected by the *agency* of the *nerves*, produces constant extrication of caloric: again, in the lungs that carbon is thrown off and united with oxygen, during which *caloric* is again *set free;* so that we have in the LUNGS a CHARCOAL FIRE constantly burning, and in the OTHER PARTS a WOOD FIRE, the one producing *carbonic acid gas*, the other *carbon;* the *food supplying*, through the circulation, the vegetable or animal *fuel* from which the *charcoal* is *prepared* that is *burned* in the *lungs.*" (pp. 42, 43.)

The following short extracts from the section upon inflammation will serve to give some idea of our author's views upon that subject, and the influence which the nerves exercise upon the contractile power of the capillaries:

"The action of the arteries is also acknowledged to be contraction, whether considered muscular or not; but there is some difference of opinion as to the degree of action of the arteries in inflamed parts. It is very common to say, that in INFLAMMATION there is an increase of arterial action; but a consideration of the phenomena, and of the nature of arterial action, will show that in inflamed parts the capillary arteries are weaker in their action; that there is diminished arterial action, for the action of arteries is contraction: now the arteries in inflamed parts are evidently larger than before—less contracted, that is, acting less."

"Let us see how far we can go in proving that the *capillaries depend* upon *nervous influence* for their contractile action. Blushing is, perhaps, the most unequivocal proof that an alteration in the nerves is the cause of sudden dilatation of the capillaries. It is not the action of the heart alone which causes the partial blush; for, first, the heart often acts stronger without causing blushing, and, next, the blush is partial; whereas, when the mere action of the heart causes increased redness of the skin, as from exercise, it is not partial, as it is in blushing from mental emotion. And this, which is sudden weakness of the capillaries, has been commonly attributed to the 'increased arterial action,' and 'determination to the face.' I attribute this giving way of the capillaries to derivation of the nervous influence, which, being directed to or expended in the brain more freely by mental emotion, robs, for the moment, the capillaries of the face of their energy."

"What is called the blush of inflammation may be brought on in a part by reiterated strong electric sparks. It may be said that the effect of the electricity is on the tissue of the capillaries; but the first effect produced is pain, showing that the operation of the electricity commences on the nerves, sensitive as well as organic." (pp. 44, 49.)

The whole of his remarks upon the subject of inflammation, with the treatment of wounds, ulcers, and suppurating surfaces, are highly interesting, and, if carefully studied and stored up in the memory, will be found to be of immense advantage, not only to the student, but to the practitioner of many years' standing; for, in the language of the author,

"Many persons of great experience practise well empirically without much brains or reasoning; but he who begins upon principle, and then profits by experience, must become a much more skilful practitioner. How many persons apply a poultice to an ulcer with a tolerable certainty of improving it, without ever knowing or caring for the rationale of the effect."

As we have already nearly reached the limits assigned us, we must content ourselves with giving a few more extracts, selected almost at random—a mode of selection, from pages so interesting, as good, perhaps, as any other. The following remarks upon rheumatism will be read with interest:

"There are forms of rheumatism which it is extremely difficult, if at all possible, to distinguish from neuralgia; so direct is the connexion between the nerves and rheumatic disease. Indeed, I believe that all rheumatism commences with lesion of the nerves of the part: to which it may be objected that in *acute* rheumatism the other tissues are red and swollen. Even so; the process of inflammation begins by functional, if with no physical derangement, in the nerves, and spreads, owing to the diffusion of the nervous tissue, through the cellular tissue of the skin, muscles, sheaths of tendons, pericardium, &c. This affords an explanation of the phenomena, and of the utility of the remedies employed: many cases are curable by tonics, while most are relievable at all stages by narcotics; some imperatively requiring bleeding, a few injured by it: the perception of which last-mentioned fact was the reason that bleeding was quite out of fashion at Edinburgh when I visited it; so much so, that patients with their wrists and ankles swollen, and the chest oppressed, were not bled, because, as was truly asserted, bleeding *sometimes* caused the convalescence to be tedious. No doubt this happened when discrimination was not used. All my life I have seen men prescribing for disease by name: one man bleeds always in acute rhumatism, another never, or scarcely ever; one gives emetics and bark in all cases, another mercury, another colchicum, &c.; one man finds digitalis of great use in rheumatism, another stares at his assertion.

"From what has been already said upon the subject of inflammation, it may be deduced why and where these remedies suit; why, for example, digitalis and bleeding will procure sleep better than opium when the brain is plethoric and feverish; and, on the other hand, that wine and other products of fermentation will procure sleep better than even opium when there is a state of inanition.

"I must beg pardon of my senior readers whilst I set down a few directions for the juniors, in further explanation.

"Do not neglect to bleed in acute rheumatism (rheumatic fever) if, with a plethoric appearance, you have the external redness and tenderness, combined with oppression at the chest, indicating tendency to pericarditis, or if there be symptoms of meningitis.

"Do not bleed in acute rheumatism, unless emetics and other treatment have failed, if there be *only external* pain and swelling, and a patient not strong, lest you have a *slow convalescence*, as any one may expect if a patient be bled unnecessarily.

"But, on the other hand, always have the fear of internal inflammation, 'metastasis,' in your mind's eye, or you may have *no convalescence* at all, but death for want of bleeding. If your patient gets severe pericarditis, you will either have death soon with fever, as I have known happen from neglect of bleeding, or a lingering death from adhesions and enlargement of the heart (such as may be seen on the shelves of every maseum), with dropsy.

"Never neglect, in acute rheumatism, to examine the chest, and inquire about it; for the patient will scarcely ever complain of it, even when affected, in consequence of his attention being drawn off to the severe pain in the limbs and muscles of the trunk. Conviction has been forced upon the medical world, that in rheumatic fever the heart uniformly participates in the disease, though 'metastasis' to the heart is not said to have taken place until symptoms of serious lesion of the organ are evinced; but the peculiar pulse of rheumatic fever declares the state of the organ. It is irritated sympathetically as in other acute diseases, hence the pulse is more frequent; but being itself affected by the rheumatism of its fibro-serous tissue, it is weakened in its action; hence it does not empty itself, causing the full soft pulse—full, because there is much blood, and soft, because the action is not energetic.

"In referring rheumatic inflammation to the nerves, I have only gone a step further than some who call certain kinds of it neuralgia. The exciting cause of rheumatism is usually cold and damp together, not either alone; intense cold will seldom produce rheumatism if the atmosphere be dry; but if the skin have been perspiring previously, so that it or the inner garment is damp, rheumatism results; warm damp does not produce it. A cold fog or rain will produce the effect, though the person has not been previously perspiring. Moisture appears to exercise a peculiar effect on the electric state of the nerves; but if the parts be warm, that is counteracted; the cold and damp together are noxiously sedative. Every person can recollect illustrations. The softer and more vascular tissues are not so easily contracted to a noxious degree as the dense fibrous tissue; hence the latter is the first to suffer from the sedative damp and cold." (pp. 251—254).

Erysipelas, he thinks, is seated in the same tissue.

"Erysipelas also commences in the nerves. Cold air does not produce the inflammation, unless the part have been previously warm and damp, and then a continued stream of cold air will produce erysipelas. Slight erysipelas of the face or ear is frequently produced in this way, and called by the peasants a blight or blast, &c." (p. 254.)

After some remarks upon the treatment of this disease, the author sums up the whole matter in these words:

"Here, in two words, is the epitome of the treatment of erysipelas—emetics and tonics. Tartar emetic, to check the inflammation, which being one of a debilitated diathesis, though benefitted by leeching or bleeding at the moment, much evacuation will even increase the tendency of the disease to return, as is known by experience. Tonics, including calomel, take away that tendency or disposition of the disease to return; and the latter by its tonic effect upon the liver, promotes the digestion and consequently the strength of the patient, who must be well nourished after the paroxysm. Practitioners well know that it is the nature of erysipelas to return periodically like gout, or even more frequently, and thus undermine the constitution. I can assure them from experience, that the plan I have laid down, persevered in, and modified according to circumstances, will eradicate it." (pp. 258, 259.)

We cannot close our notice without requesting all, who have an opportunity, to read this work carefully, and we doubt not they will consider themselves well paid for their trouble. The book, we admit, has faults both in the style and matter, but its good qualities so far out-number them, that we feel ourselves fully justified in recommending it to the profession. Works of this character are calculated to inspire the members of the profession with more confidence in the principles of the healing art, and to check that spirit of *quackery* which at this time seems to pervade the whole civilized world—if we may be allowed to judge from the vast amount of patent medicines and secret nostrums which are manufactured and consumed. That quackery must exist so long as disease exists, there is much reason to believe; but, at the same time, much may be done to check it by a proper spirit of determination on the part of physicians to resist its progress. The use of secret remedies does not meet that opposition from the profession which a just regard for its own honor and the good of mankind require. We should make it a rule never to countenance their exhibition in any manner or form—and a general determination of this kind would be productive of much good, and lessen this crying evil. How often do we witness the names of persons who write themselves "M.D.," signed to certificates lauding the efficacy of some patent mixture of whose ingredients they are ignorant. And it is even said, we know not with what truth, that a professor in an American medical school has recently been selling a secret remedy. It is hoped, for the honor of the profession, that this statement is not true.* Physicians are often deterred, by false notions of delicacy and friendship, from opposing the use of these remedies—one dislikes to injure the feelings of a friend, who assures him that *this* or *that* is an excellent article and has done wonders in the way of cure—but the most frequent cause, perhaps, of this apathy, is their regard for the apothecaries with whom they are acquainted, and who have large amounts of these articles for sale. Such we know to be the case in the country, and have reason to believe it to be the case in the cities also. This spirit, though originating in the best feelings of our nature, should be thrown aside when brought in competition with the general welfare; and the profession should come up as one man, and at all times and on every occasion condemn quackery in every form.

R. H.

---

*It is too true. He holds an important chair in a Kentucky School of Medicine.—C.

